# Decrease of myofiber branching via muscle-specific expression of the olfactory receptor mOR23 in dystrophic muscle leads to protection against mechanical stress

**DOI:** 10.1186/s13395-016-0077-7

**Published:** 2016-01-21

**Authors:** Christophe Pichavant, Thomas J. Burkholder, Grace K. Pavlath

**Affiliations:** 1 Department of Pharmacology, Emory University, Atlanta, GA USA; 2 School of Applied Physiology, Georgia Institute of Technology, Atlanta, GA USA; 3 Present address: Department of Genetics, Stanford University, Stanford, CA USA; 4 1510 Clifton Road, Room 5024, Atlanta, GA 30322 USA

**Keywords:** mOR23, Myofiber branching, Mechanical stress, Muscular dystrophy, Muscle regeneration

## Abstract

**Background:**

Abnormal branched myofibers within skeletal muscles are commonly found in diverse animal models of muscular dystrophy as well as in patients. Branched myofibers from dystrophic mice are more susceptible to break than unbranched myofibers suggesting that muscles containing a high percentage of these myofibers are more prone to injury. Previous studies showed ubiquitous over-expression of mouse olfactory receptor 23 (mOR23), a G protein-coupled receptor, in wild type mice decreased myofiber branching. Whether mOR23 over-expression specifically in skeletal muscle cells is sufficient to mitigate myofiber branching in dystrophic muscle is unknown.

**Methods:**

We created a novel transgenic mouse over-expressing mOR23 specifically in muscle cells and then bred with dystrophic (*mdx*) mice. Myofiber branching was analyzed in these two transgenic mice and membrane integrity was assessed by Evans blue dye fluorescence.

**Results:**

mOR23 over-expression in muscle led to a decrease of myofiber branching after muscle regeneration in non-dystrophic mouse muscles and reduced the severity of myofiber branching in *mdx* mouse muscles. Muscles from *mdx* mouse over-expressing mOR23 significantly exhibited less damage to eccentric contractions than control *mdx* muscles.

**Conclusions:**

The decrease of myofiber branching in *mdx* mouse muscles over-expressing mOR23 reduced the amount of membrane damage induced by mechanical stress. These results suggest that modifying myofiber branching in dystrophic patients, while not preventing degeneration, could be beneficial for mitigating some of the effects of the disease process.

**Electronic supplementary material:**

The online version of this article (doi:10.1186/s13395-016-0077-7) contains supplementary material, which is available to authorized users.

## Background

Duchenne muscular dystrophy (DMD) is an X-linked disease due to the absence of dystrophin in muscle [1] that affects about one in every 3500 boys [2]. The lack of dystrophin in DMD patients leads to progressive muscle degeneration and weakness resulting in death from heart or respiratory failure during the third decade of life. There is currently no curative treatment for this disease. One characteristic of dystrophic muscle is the presence of myofibers with an abnormal branching cytoarchitecture [3, 4]. These aberrant myofibers called branched myofibers or split myofibers contain one or more offshoots of daughter myotubes contiguous with the parent myofiber.

Myofiber branching negatively impacts dystrophic muscles as demonstrated using extensor digitorum longus (EDL) muscles from dystrophin-deficient *mdx* mice. Unbranched and branched myofibers isolated from *mdx* EDL muscles were subjected to Ca^2+^-force activation. Branched myofibers broke before reaching maximal calcium activation, whereas unbranched myofibers were able to sustain maximal force without any break [5]. When EDL muscles from *mdx* mice were eccentrically contracted (EC), then incubated in Evans blue dye (EBD), approximately 70 % of the isolated branched myofibers showed EBD uptake at branch points indicating membrane damage, while no dye was observed in unbranched myofibers [5]. Following Ca^2+^ osmotic stress, branched myofibers from *md*x muscles also exhibited a higher number of osmotic-induced Ca^2+^ sparks than unbranched myofibers [6]. Together, these data indicate that myofiber branching impairs multiple aspects of muscle physiology in dystrophic muscles. This leads to the question of how to reduce myofiber branching in dystrophic muscles in order to improve muscle physiology.

We recently identified mouse olfactory receptor 23 (mOR23, olfr16) as a molecule regulating myofiber branching in mouse muscles [7]. Olfactory receptors (OR) are G protein-coupled receptors mainly found in neurons of the olfactory epithelium where they have been extensively studied, but they are also expressed in non-olfactory tissues such as the brain, tongue, testis, spleen, prostate, kidney, and smooth and skeletal muscle [8, 9]. OR serve as specialized chemosensors and are activated by exogenous ligands known as odorants in olfactory neurons or by endogenous ligands in non-olfactory tissues. Endogenous OR ligands have only been identified in non-olfactory tissues for olfr78 in the carotid body, kidney, peripheral vasculature, and prostate [9–11]. Mouse muscles electroporated with a plasmid expressing mOR23 under the control of a ubiquitous promoter contained fewer branched myofibers and fewer branches per myofiber [7]. In the skeletal muscle, several types of cells are important for muscle regeneration such as neutrophils [12], macrophages [13], and fibroblasts [14] in addition to muscle stem cells [15]. Since mOR23 was ubiquitously over-expressed, whether mOR23 was required specifically in muscle cells to regulate myofiber branching could not be addressed in these experiments. To directly address the role of mOR23 in muscle cells during myofiber branching, we created a novel transgenic mouse over-expressing mOR23 under the control of a specific muscle promoter [16]. In-depth quantitative analyses of myofiber branching in these transgenic mice demonstrated that myofiber branching was reduced after chemically induced muscle injury. Subsequently, these mice were bred with *mdx* mice to determine whether muscle-specific over-expression of mOR23 was also beneficial in the context of repeated cycles of muscle degeneration/regeneration. We observed decreased myofiber branching in *mdx* mice over-expressing mOR23 resulting in less membrane damage of the dystrophic muscles after mechanical stress. Together, these results indicate that expression of mOR23 specifically in skeletal muscle is sufficient to decrease the incidence of myofiber branching after muscle injury.

## Methods

### Mice

Wild type (C57BL/6) and *mdx* (C57BL/10) mice were purchased from The Jackson Laboratory (Bar Harbor, ME, USA). To create mOR23 transgenic mice, the mOR23 gene was excised from pME-18S [17] using XbaI and BamHI restriction enzymes and ligated into pBSX-HSAvpA [18] previously cut with NotI and XbaI restriction enzymes. The resulting plasmid was named pHSA.mOR23 and cut using PvuI and KpnI restriction enzymes. The subsequent fragment was used by the Emory Transgenic Core to produce two independent lines of mOR23 transgenic mice, TG1 and TG2. TG1 was bred with *mdx* mice to create transgenic *mdx* mice over-expressing mOR23 (Tg-*mdx*). Female and male mice were used in all experiments at 8–12 weeks of age unless described otherwise. Experiments involving animals were performed in accordance with approved guidelines and ethical approval from Emory University’s and Georgia Institute of Technology’s Institutional Animal Care and Use Committees.

### Quantitative real-time PCR

Total RNA from gastrocnemius muscles was isolated using TRIzol reagent (Invitrogen, Carlsbad, CA, USA) according to the manufacturer’s protocol. For each sample, 500 ng of RNA was digested with DNAseI (Invitrogen) to remove any DNA contaminants and cDNA synthesis was performed by M-MLV reverse-transcriptase (Invitrogen). Using a real-time (RT) PCR system (StepOnePlus, Applied Biosystem, Foster City, CA, USA), the relative levels of mOR23 were determined by the ΔΔCt method [19] and normalized to the housekeeping gene HPRT1. Primers were purchased from SA Biosciences (Valencia, CA, USA) (mOR23: PPM60724B, HPRT1: PPM03559E). All reactions were performed in duplicate.

### Muscle injury

#### BaCl_2_

Mice were anesthetized with an intraperitoneal injection of a solution containing 80 mg/kg ketamine HCl/5 mg/kg xylazine and subcutaneously injected with an analgesic (0.1 mg/kg buprenorphine) before and after muscle injury. Injury was induced in gastrocnemius muscles of anesthetized mice by injection of 40 μl of 1.2 % BaCl_2_ [20]. Muscles were collected 2–3 weeks after the injury and either snap frozen in liquid nitrogen or enzymatically digested for isolation of single myofibers.

#### Eccentric contraction

The day before the eccentric contraction protocol, mice were intraperitoneally injected with EBD (Sigma-Aldrich) (100 μL of 1 % EBD in PBS per 10-g body weight). The following day, mice were anesthetized by intraperitoneal injection of a ketamine/xylazine cocktail (90 mg/kg ketamine, 15 mg/ml xylazine), with supplemental doses as necessary. The left plantaris and soleus muscles were ablated and a 4-0 suture tied around the Achilles tendon at the calcaneus. The insertion of the tendon was clipped from the calcaneus, leaving a small bone chip to anchor the suture. The knee was immobilized by a spring clamp fixed to the femoral condyles, and the gastrocnemius complex was attached to a force-reporting servo motor (Aurora Scientific, Aurora, ON, Canada). The sciatic nerve was exposed and mounted on bipolar stainless steel hook electrodes. A series of twitch contractions was used to determine the stimulation voltage and muscle length (*L*_0_) required for maximal active force production. Muscles were stimulated using constant current, determined for each animal as twice the current required (700–1500 uA) to yield maximum twitch force. *L*_0_ was determined from twitches by adjusting the muscle length to maximize active tension. The lengthening activation protocol consisted of 300 stimulations, each 500 ms of 0.1-ms pulses delivered at 70 Hz and 2× maximal voltage. The muscle was held isometric for 200 ms at *L*_0_ − 10 % optimal fiber length (*L*_f_), stretched to *L*_0_ + 10 % *L*_f_ over 200 ms, and held for 200 ms. The muscle was returned to *L*_0_ − 10 % *L*_f_ after relaxation with 500-ms rest between activations. At the end of the protocol, muscles were collected and snap frozen in liquid nitrogen.

### Single myofiber isolation

Single myofibers were isolated from gastrocnemius muscles as previously described [20]. Briefly, muscles were gently dissected, added to a tube containing 4000 U collagenase type I (Worthington Biochemical, Lakewood Township, NJ, USA). The tube was rocked at 26 rpm in an Enviro-Genie (Scientific Industries, Bohemia, NY, USA) set at 37 °C. Wild type muscles were digested for 90 min and for up to 120 min for *mdx* and Tg-*mdx* muscles. After incubation in collagenase, single myofibers were washed and transferred into a Matrigel (BD Pharmigen, San Diego, CA, USA)-coated 24-well plate using a fire-polished Pasteur pipette. Isolated myofibers were allowed to settle for 30 min in the well before the plates were centrifuged at 1100*g* for 20 min and then fixed with 3.7 % formaldehyde for 10 min. A total of 471 and 475 myofibers were isolated for *mdx* and Tg-*mdx* mice, respectively, and between 496 and 828 for wild type ( WT), TG1, and TG2 BaCl_2_-injured mice.

### Single myofiber analysis

Single myofibers were stained with 1 μg/ml 4′,6-diamidino-2-phenylindole (DAPI, Sigma-Aldrich, St Louis, MO, USA) for 1 min to visualize nuclei. Myofibers were visualized using an Axiovert 200M microscope (Carl Zeiss Microscopy, Thornwood, NY, USA), and images were acquired using a 10× or 20× Plan-Neofluar objective (Carl Zeiss Microscopy) and camera (QImaging, Surrey, BC, Canada) with OpenLab 5.50 software (PerkinElmer, Waltham, MA, USA). All images were uniformly processed for size, brightness, and contrast using Photoshop CS6 (Adobe Systems, San Jose, CA, USA). Myofibers with at least four centrally located nuclei in a row were considered regenerated. Branches were grouped into three morphologic categories: bifurcated, split, and process although the significance of these different types of branches is not well understood [6, 20, 21].

### Histological analysis

Serial 12-μm cryostat sections were obtained throughout frozen muscles. For histological analyses, sections were stained with hematoxylin (Thermo Fisher Scientific, Waltham, MA, USA) and eosin (Sigma-Aldrich) stained sections using ImageJ (NIH, Bethesda, MD, USA). For all tissue section studies, three to four representative sections imaged at ×200 magnification were analyzed from gastrocnemius muscles. For EBD quantification, images were acquired from the same anatomic region in three to four different sections and the EBD fluorescence of each image was measured as pixel intensity using Image J. All images were acquired using an Axioplan microscope with a 0.5 NA 20× Plan-Neofluar objective (Carl Zeiss Microscopy) and charge-coupled device camera (Carl Zeiss Microscopy) with Scion Image 1.63 (Scion Corporation, Frederick, MD, USA). All images were uniformly processed for size, brightness, and contrast using Photoshop CS6 (Adobe).

### Statistical analyses

To determine statistical significance for two groups, comparisons were made using an unpaired or paired Student’s *t* test. Chi-square tests were performed for the analysis of the number of branches per myofiber. The significance of results from multiple groups was evaluated by a one-way analysis of variance (ANOVA) with Bonferroni’s post-test. The Friedman test with Dunn’s post-test was used to analyze EBD fluorescence in muscle sections. Statistical analyses were performed using GraphPad Prism v.6 (GraphPad Software, La Jolla, CA, USA). A *p* value <0.05 was considered significant.

## Results

### mOR23 transgenic mice

**Fig. 1:**
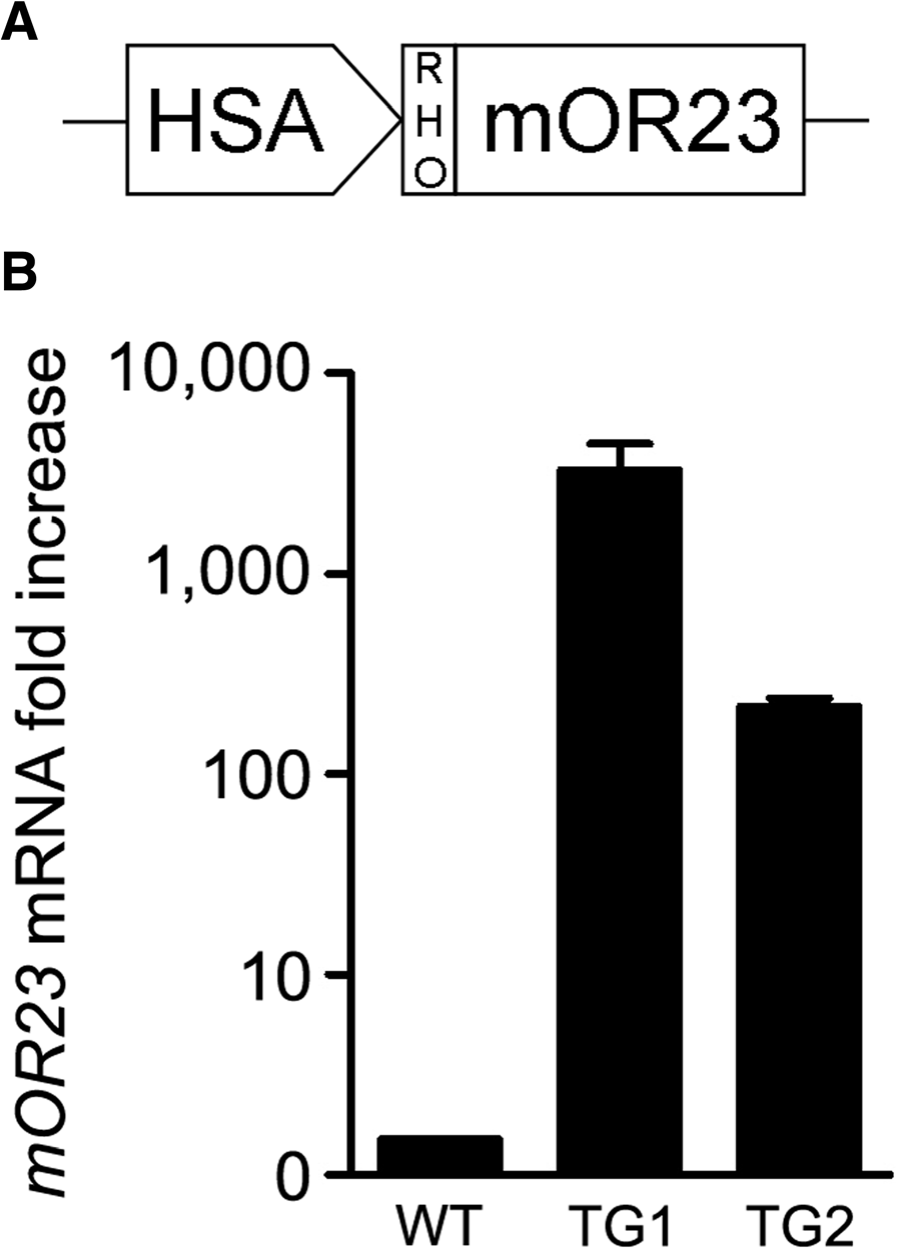
mOR23 expression in transgenic mouse muscles. **a** Schematic representation of the construct used to drive muscle-specific expression of mOR23. mOR23 was fused with rhodopsin (RHO) to increase mOR23 cell surface expression. *HSA* human α-skeletal actin. **b** *mOR23* RNA levels were greatly increased in the gastrocnemius muscles of two different transgenic (TG) mouse lines compared to wild type (WT): TG1 (3302 ± 1141), TG2 (218 ± 21). Data are mean ± SEM with *n* = 4–6 for each genotype

To determine the role of mOR23 in regulating myofiber branching specifically in muscle cells, we created a novel transgenic mouse over-expressing mOR23 under the control of the human skeletal actin (HSA) promoter, a muscle-specific promoter (Fig. 1a). Two independent lines of transgenic mice (TG1 and TG2) were produced and tested for mOR23 over-expression. As shown in Fig. 1b, *mOR23* messenger RNA (mRNA) was over-expressed in both lines compared to control mice (WT). Since the levels of *mOR23* mRNA in TG1 were more than 10 times the levels found in TG2, we mainly focused our subsequent experiments on TG1.

### Analysis of myofiber branching in isolated myofibers

**Fig. 2:**
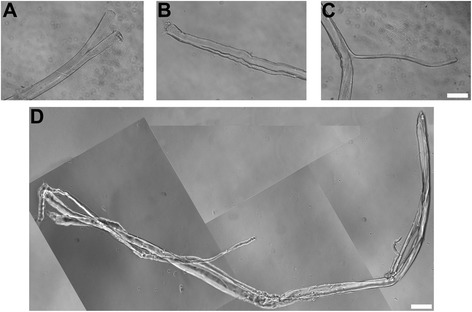
Phase-contrast image of branched myofibers. **a**–**c** Phase-contrast images of the three branch types studied: bifurcated (**a**), split (**b**), and process (**c**). *Bar* = 100 μm. **d** Phase-contrast image of a highly branched myofiber from a 15-week-old *mdx* mouse. *Bar* = 250 μm

To compare myofiber branching, single myofibers were analyzed rather than transverse sections of muscle tissue since all branches along the length of isolated myofibers can be easily identified. Single myofibers were isolated from enzymatically digested gastrocnemius muscles, then fixed and stained with DAPI to visualize nuclei. Myofibers were examined using phase contrast and fluorescence microscopy. Myofibers with at least four centrally located nuclei in a row were scored as regenerated. Branches were grouped into three morphologic categories (Fig. 2a–c): bifurcated, split, and process as previously described [20]. Myofibers may have multiple branch types as is frequently encountered in aged *mdx* muscles (Fig. 2d).

### Muscle-specific expression of mOR23 decreases myofiber branching in chemically injured mouse muscles

**Fig. 3:**
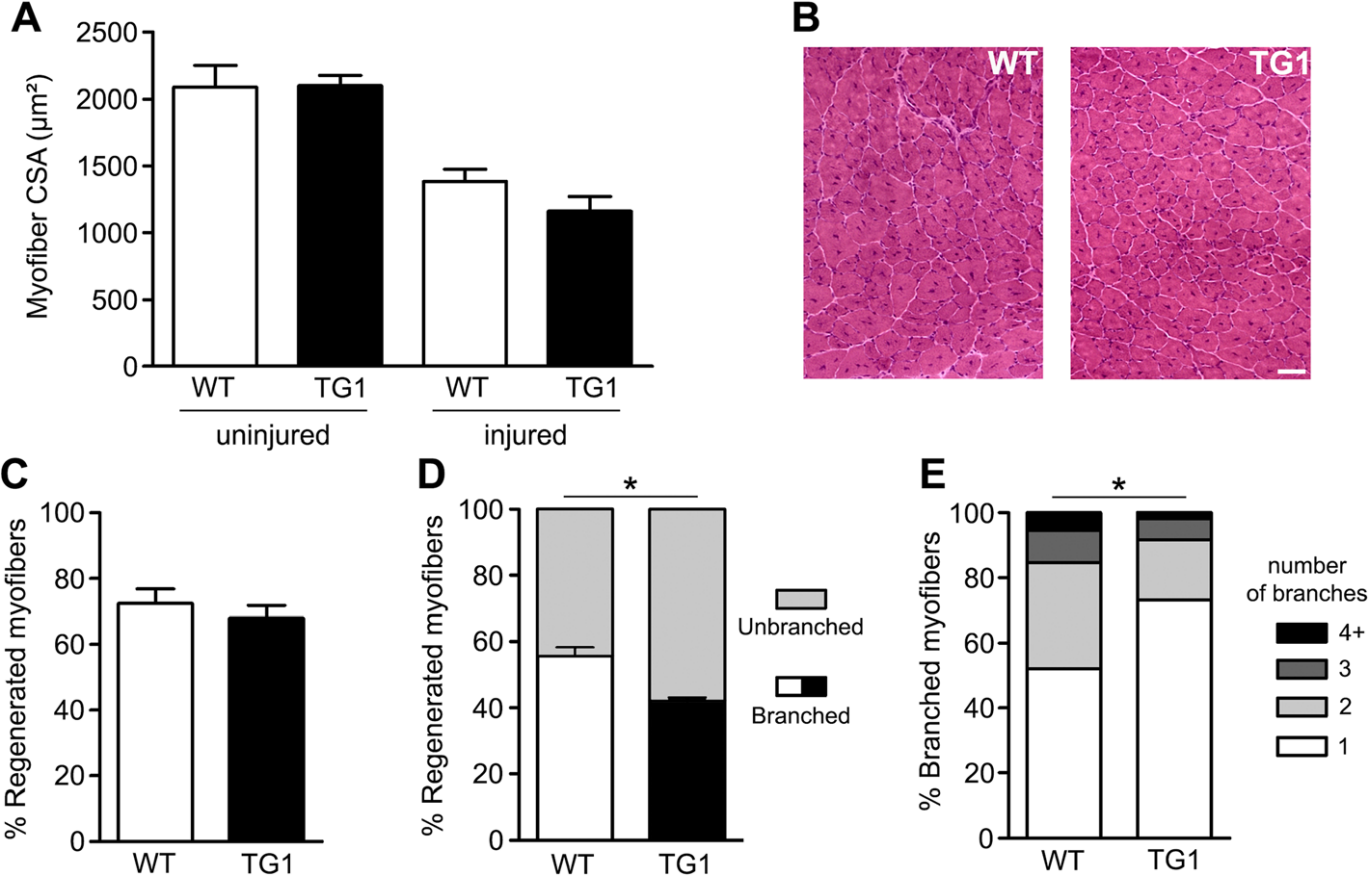
mOR23 over-expression decreases myofiber branching after muscle regeneration. **a** Myofiber cross-sectional areas (CSA) of WT and TG1 gastrocnemius muscles were comparable before injury and after 2 weeks of regeneration. **b** Representative images of injured WT and TG1 muscles after 2 weeks of regeneration. *Bar* = 100 μm. **c** The percentage of regenerated myofibers was not significantly different in WT and TG1 muscles 3 weeks after muscle injury but TG1 muscles contained significantly less branched regenerated myofibers than WT muscles (**d**). **e** The number of branches per branched myofiber was also significantly decreased in injured TG1 muscles compared to WT (chi-square = 21.6, *df* = 3). *n* = 94–196 myofibers isolated per genotype and mouse. Data are mean ± SEM and *n* = 4–6 mice for each genotype with *p < 0.05

Under basal conditions in young mice, very low levels of myofiber branching (1 %) occur in muscle but the frequency increases dramatically in response to muscle regeneration [7, 20, 22–24]. Therefore, we determined whether mOR23 over-expression in muscle cells was sufficient to regulate muscle regeneration and myofiber branching in chemically injured mouse muscles. To assess whether mOR23 protein would be over-expressed in differentiating TG1 muscle cells during regeneration, we analyzed muscle cells in vitro so as to assay mOR23 specifically in muscle cells in the absence of other cell types. mOR23 protein levels were increased by ~40 % in TG1 compared to WT at 48 h of differentiation (Additional file 1: Figure S1). Subsequently, gastrocnemius muscles of WT and TG1 were injured using barium chloride (BaCl_2_) to determine whether the muscle-specific over-expression of mOR23 affected muscle regeneration. We first analyzed the myofiber cross-sectional area (CSA) of uninjured and injured gastrocnemius muscles. Two weeks after injury, no significant difference in myofiber CSA was observed between WT and TG1 muscles (Fig. 3a, b) suggesting similar overall muscle regenerative ability. To analyze myofiber branching, single myofibers were isolated from muscles 3 weeks post-injury. As a control, myofibers were also isolated from uninjured muscles. The same number of branched myofibers (~1 %) was found in uninjured WT or TG1 muscles (data not shown). No significant difference in the percentage of regenerated myofibers was observed between WT and TG1 (Fig. 3c) indicating a similar amount of muscle damage in both mice. Remarkably, the percentage of branched regenerated myofibers and the number of branches per branched myofiber were significantly decreased in TG1 compared to WT (Fig. 3d, e). No significant difference was observed among the three types of branches between WT and TG1 (Additional file 2: Figure S2). To validate these results, we also analyzed myofiber branching in the gastrocnemius muscles of TG2 mice after muscle injury. Similar to the results obtained with TG1, we observed no difference in the percentage of regenerated myofibers between WT and TG2 (Additional file 3: Figure S3A) but the percentage of branched regenerated myofibers was decreased in TG2 (Additional file 3: Figure S3B). However, the number of branches per branched myofiber was not significantly different between WT and TG2 (Additional file 3: Figure S3C) as it was for TG1 (Fig. 3e). This discrepancy in the number of branches between the two transgenic mouse lines may be due to the fact that *mOR23* mRNA levels were 10 times higher in TG1 than in TG2 (Fig. 1b). Overall, these data indicate that mOR23 expression in muscle cells is sufficient to regulate myofiber branching during muscle regeneration.

### mOR23 over-expression reduces myofiber branching in dystrophic mouse muscles

**Fig. 4:**
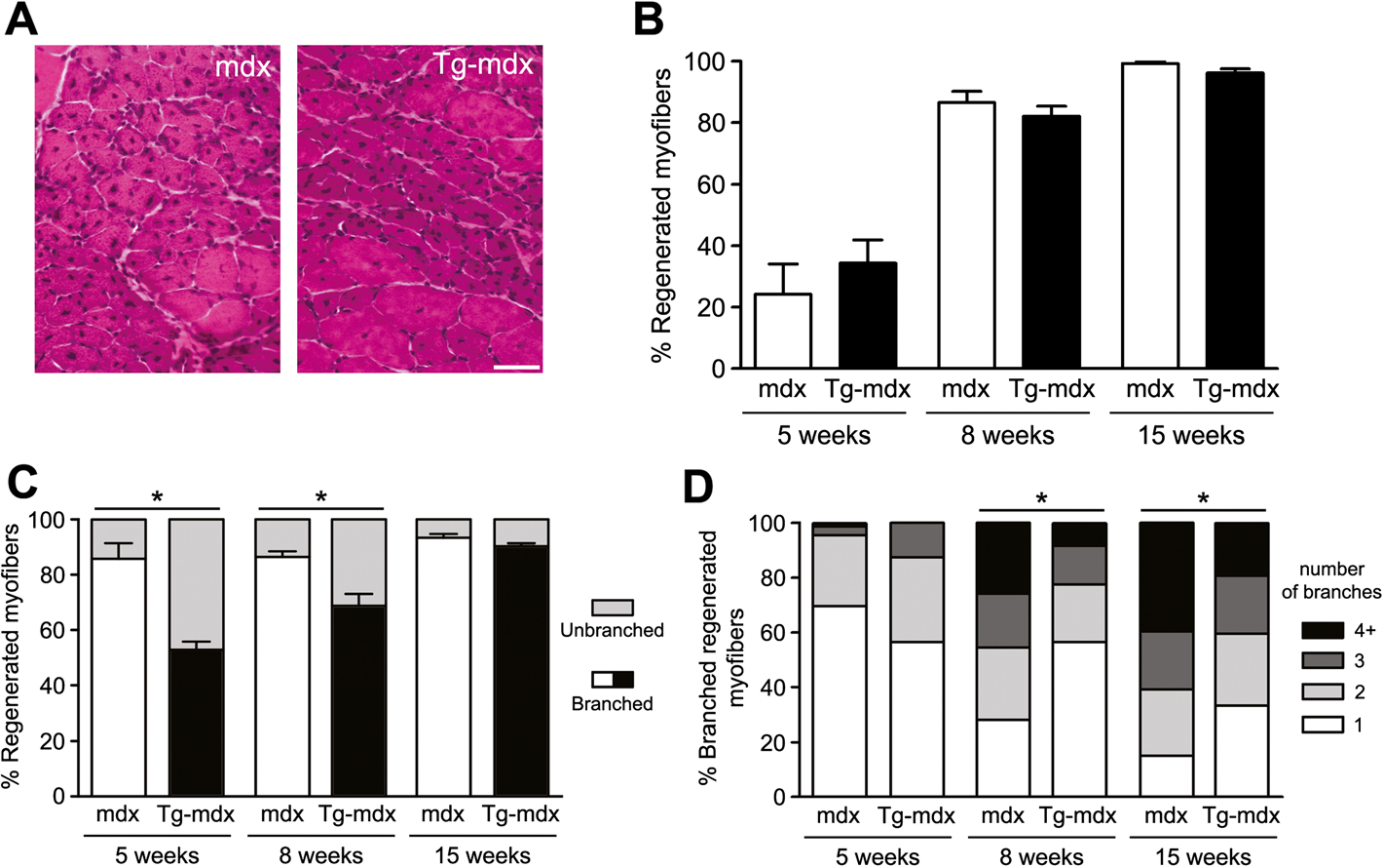
Myofiber branching is decreased in *mdx* muscles over-expressing mOR23. **a** Representative images of 8-week-old *mdx* and Tg-*mdx* gastrocnemius muscles. *Bar* = 100 μm. **b** The percentage of regenerated myofibers was not significantly different between *mdx* and Tg-*mdx* muscles at the three ages (5, 8, and 15 weeks) studied. **c** Tg-*mdx* muscles had significantly less branched regenerated myofibers than *mdx* muscles at 5 and 8 weeks of age. **d** The number of branches per branched myofiber was also significantly decreased in Tg-*mdx* muscles compared to *mdx* at both 8 weeks (chi-square = 12.3, *df* = 3) and 15 weeks of age (chi-square = 13.9, *df* = 3). *n* = 32–103 myofibers isolated per age and mouse. Data are mean ± SEM and *n* = 3–4 for each genotype at each age with *p < 0.05

We next bred TG1 with *mdx* mice to create Tg-*mdx* to determine whether muscle-specific over-expression of mOR23 would also decrease myofiber branching in the context of the repeated cycles of muscle degeneration/regeneration found in *mdx* mice [25, 26]. The histology of gastrocnemius muscles from *mdx* and Tg-*mdx* mice were first compared. For both genotypes at 8 weeks of age, a similar heterogeneity in myofiber sizes was observed in muscle sections indicating no gross difference in muscle regenerative ability (Fig. 4a). Since we previously demonstrated that the percentage of branched myofibers in gastrocnemius muscles rapidly increased in *mdx* mice between 3 and 10 weeks of age reaching ~90 % [20], single myofibers from gastrocnemius muscles of *mdx* and Tg-*mdx* mice were analyzed at 5, 8, and 15 weeks of age. At each age, the percentage of regenerated myofibers was not significantly different between both genotypes (Fig. 4b) indicating a similar amount of muscle damage occurred in both dystrophic lines. The percentage of regenerated myofibers that were branched was significantly decreased in Tg-*mdx* at 5 and 8 weeks of age but not at 15 weeks of age (Fig. 4c). Interestingly, the number of branches per branched myofiber was significantly reduced in 8- and 15-week-old Tg-*mdx* compared to *mdx* mice but not at 5 weeks of age. These results suggest that at 5 weeks of age when a low amount of damage is occurring in dystrophic muscles [25, 26], mOR23 over-expression only acts to decrease the likelihood of a myofiber becoming branched. In contrast, at 15 weeks of age when severe muscle degeneration already occurred in *mdx* mice [25, 26], mOR23 over-expression cannot influence whether a myofiber becomes branched or not but can decrease the number of branches that arise per damaged myofiber. At the three ages analyzed, the three types of branches were globally equally distributed between *mdx* and Tg-*mdx* (Additional file 4: Figure S4). Overall, these findings demonstrate that mOR23 over-expression does not protect against muscle degeneration induced by the lack of dystrophin but can reduce the severity of myofiber branching arising in dystrophic muscles.

### mOR23 over-expression protects dystrophic muscle against mechanical damage

**Fig. 5:**
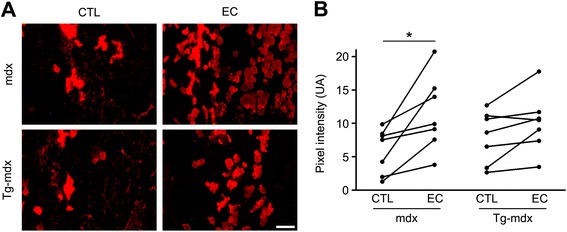
Over-expression of mOR23 in *mdx* muscles protects against membrane injury after eccentric muscle contractions. **a** Representative images of gastrocnemius muscles from *mdx* and Tg-*mdx* mice which underwent eccentric contraction (EC) or not (CTL, contralateral muscle). Evans blue dye (EBD) fluorescence identified myofibers with increased membrane permeability. *Bar* = 100 μm. **b** *mdx* muscles were significantly more permeable to EBD after EC than Tg-*mdx* muscles. Each *line* represents the CTL and EC muscle of one mouse, *n* = 7 for each genotype with *p < 0.05

Branched *mdx* myofibers are more fragile when subjected to mechanical stress than unbranched *mdx* myofibers [5, 6, 21, 27, 28]. Since Tg-*mdx* mice displayed less severe myofiber branching than control *mdx* mice, we subsequently assessed the membrane integrity of Tg-*mdx* and *mdx* mouse muscles in response to in vivo mechanical stress. Eight-week-old mice were used in this experiment since both the percentage of branched myofibers and the number of branches per myofiber were significantly decreased in Tg-*mdx* myofibers compared to *mdx*. To determine changes in membrane integrity in response to mechanical stress, we used Evans blue dye (EBD) as an in vivo marker of myofiber damage as EBD penetrates into myofibers in response to disruption of the sarcolemma [5]. Damaged myofibers are then readily identifiable by the fluorescence emitted by the dye. To induce muscle injury, gastrocnemius muscles were subject to 300 eccentric contractions (EC) of 10 % optimal fiber length. Peak isometric force was similar in *mdx* (11.3 ± 3.2 N/g) and Tg-*mdx* (8.59 ± 4.2 N/g, *p* = 0.22), and lengthening resulted in similar eccentric forces (*mdx* 15.4 ± 4.1 N/g; Tg-*mdx* 12.4 ± 6.2 N/g; *p* = 0.35). Repeated activation resulted in dramatic and rapid loss of contractile capacity, with declines of 85 ± 4.9 % in *mdx* and 74 ± 18 % in Tg-*mdx* after the first 100 activations (*p* = 0.19 between genotypes). Muscles were collected 30 min after the last contraction to let the dye penetrate damaged myofibers. Muscle sections of control contralateral and EC muscles were then visualized for EBD fluorescence in both genotypes (Fig. 5a), and the pixel intensity was quantified (Fig. 5b). Since muscle physiology varies considerably between animals, we compared EBD pixel intensity between contralateral and EC muscles for each mouse. We observed a higher increase of EBD pixel intensity in *mdx* muscles after eccentric contractions compared to Tg-*mdx* muscles (Fig. 5b) indicating that Tg-*mdx* muscles were more resistant to sarcolemmal damage in response to eccentric contractions These data suggest that the over-expression of mOR23 in *mdx* mice protects muscles against mechanical stress by decreasing the overall number of branches in myofibers.

## Discussion

The results reported here expand previous studies of mOR23 in muscle tissue [7] and give insights into the formation of branched myofibers, which are detrimental for normal muscle physiology. One significant finding of this study is that following acute muscle injury, myofiber branching was decreased in transgenic mice over-expressing mOR23 specifically in muscle cells. Depending on the level of mOR23 over-expression, the number of branches per branched myofiber was also reduced. These results suggest that putative endogenous ligand(s) of mOR23 [7, 29] produced in response to muscle damage were not limiting under these conditions. Another important finding is that when mOR23 was over-expressed in myofibers in a dystrophic environment with ongoing cycles of myofiber degeneration/regeneration, myofiber branching was also decreased. These are the first studies to show that myofiber branching can be decreased in dystrophic muscles. Depending on the amount of damage that occurred in dystrophic mice with age, the parameters of the myofiber branching varied. At 8 weeks of age, dystrophic muscles were characterized by a reduction in the percentage of branched myofibers as well as a decrease of the number of branches per branched myofiber. However, at 15 weeks of age only the number of branches per branched myofiber was decreased. These differences could be due to changes in the production of putative endogenous mOR23 ligand(s) or to derangements in cell signaling pathways with age in dystrophic mice [30, 31]. Taken together our data indicate that mOR23 over-expression in muscle cells is sufficient to reduce myofiber branching in mice suggesting that biologic processes acting within muscle cells are important for determining whether branches form or not upon muscle injury.

Previous studies of dystrophic muscles indicated that myofiber branching is detrimental for normal muscle physiology [5, 6, 21, 27, 28]. Branched myofibers tends to break at the branch point thereby decreasing the ability of myofibers to properly contract [5, 21]. Another key finding in our studies was that a decrease of myofiber branching in dystrophic muscle via the over-expression of mOR23 in muscle cells improved membrane integrity in response to mechanical stress induced by a stretch of eccentric contractions. These results suggest that modifying myofiber branching in dystrophic patients, while not preventing degeneration, could be beneficial for mitigating some of the effects of the disease process. Such a therapy could be used in combination with other disease-modifying strategies currently under development [32, 33].

Olfactory receptor activation leads to an increase in cyclic adenosine monophosphate (cAMP) synthesis through the activation of adenylate cyclase type III via a G protein-coupled cascade in the neurons [8], sperm [34], and kidney [35] as well as skeletal muscle [7]. In olfactory neurons, this increase in cAMP can regulate expression of adhesion molecules such as Kirrel3 or EphrinA5 [36], neuropilin-1 which plays versatile roles in angiogenesis, cell survival and migration [37], and multiple ion channels [8]. Myofiber branching has been postulated to arise from aberrant muscle cell fusion [6, 20, 21, 38]. Previous studies of mOR23 function in vitro revealed that cell migration, cell-cell adhesion, and formation of multinucleated myotubes were significantly inhibited with loss of mOR23 by small interfering RNA (siRNA) or increased by its over-expression [7] suggesting that mOR23 may regulate cell fusion through downstream migration and adhesion molecules. However, no downstream effectors of mOR23 in vitro or in vivo have been identified to date.

## Conclusions

mOR23 signaling within muscle cells may serve as an effective pharmacologic target for improving muscle architecture and physiology in dystrophic patients. The use of small molecules to activate downstream processes normally induced by mOR23 signaling in muscle cells could overcome any potential limitations in the amount of putative endogenous ligand(s) produced at different stages of the dystrophic disease process. Additional studies are needed to identify other molecules and pathways that synergize with mOR23-dependent pathways to further decrease myofiber branching in dystrophic muscles.

## Additional files

Additional file 1: Figure S1. mOR23 is over-expressed during myogenesis in TG1 myoblasts. (A) Immunoblot revealed that mOR23 levels increased after 48 h of myoblast differentiation in vitro but the levels of mOR23 were higher in TG1 muscle cells than in WT. (B) Quantification of mOR23 levels in differentiating muscle cells in vitro. Data are mean ± SEM; *n* = 3 with *p < 0.05.

Additional file 2: Figure S2. Quantification of branch types in WT and TG1. No significant difference was observed between WT and TG1 myofibers for the distribution of the bifurcated, split, and process branch types 3 weeks after muscle injury. *n* = 27–88 branched myofibers isolated per mouse. Data are mean ± SEM and *n* = 4–6 mice for each genotype.

Additional file 3: Figure S3. Myofiber branching is also decreased in mOR23 transgenic mouse line 2 after muscle regeneration. (A) The percentage of regenerated myofibers in gastrocnemius muscles was not significantly different in WT and TG2 mice 3 weeks after muscle injury but TG2 muscles had significantly fewer branched regenerated myofibers than WT muscles (B). (C) The number of branches per branched myofiber was not significantly decreased in injured TG muscles compared to WT muscles (chi-square = 2.51, *df* = 3). *n* = 108–196 myofibers isolated per genotype and mouse. Data are mean ± SEM and *n* = 4 mice for each genotype with *p < 0.05.

Additional file 4: Figure S4. Quantification of branch types in mdx and Tg-mdx. Except for a significant increase of the split branch type in *mdx* compared to Tg-*mdx* at 5 weeks of age, the types of branches did not differ between *mdx* and Tg-*mdx. n* = 7–64 branched myofibers isolated per age and mouse. Data are mean ± SEM and *n* = 3–4 for each genotype at each age with *p < 0.05.

## Abbreviations

cAMP: cyclic adenosine monophosphate
CSA: cross-sectional area
DAPI: 4′,6-diamidino-2-phenylindole
DMD: Duchenne muscular dystrophy
EBD: Evans blue dye
EC: eccentric contraction
EDL: extensor digitorum longus
HSA: human skeletal actin
mOR: mouse olfactory receptor

## References

[1] Hoffman EP, Brown RH, Kunkel LM (1987). Dystrophin: the protein product of the Duchenne muscular dystrophy locus. Cell.

[2] Bushby K, Finkel R, Birnkrant DJ, Case LE, Clemens PR, Cripe L (2010). Diagnosis and management of Duchenne muscular dystrophy, part 1: diagnosis, and pharmacological and psychosocial management. Lancet Neurol.

[3] Swash M, Schwartz MS (1977). Implications of longitudinal muscle fibre splitting in neurogenic and myopathic disorders. J Neurol Neurosurg Psychiatry.

[4] Ontell M (1981). Muscle fiber necrosis in murine dystrophy. Muscle Nerve.

[5] Head SI (2010). Branched fibres in old dystrophic mdx muscle are associated with mechanical weakening of the sarcolemma, abnormal Ca2+ transients and a breakdown of Ca2+ homeostasis during fatigue. Exp Physiol.

[6] Lovering RM, Michaelson L, Ward CW (2009). Malformed mdx myofibers have normal cytoskeletal architecture yet altered EC coupling and stress-induced Ca2+ signaling. Am J Physiol Cell Physiol.

[7] Griffin CA, Kafadar KA, Pavlath GK (2009). MOR23 promotes muscle regeneration and regulates cell adhesion and migration. Dev Cell.

[8] Kang N, Koo J (2012). Olfactory receptors in non-chemosensory tissues. BMB Rep.

[9] Pluznick JL, Protzko RJ, Gevorgyan H, Peterlin Z, Sipos A, Han J (2013). Olfactory receptor responding to gut microbiota-derived signals plays a role in renin secretion and blood pressure regulation. Proc Natl Acad Sci U S A.

[10] Neuhaus EM, Zhang W, Gelis L, Deng Y, Noldus J, Hatt H (2009). Activation of an olfactory receptor inhibits proliferation of prostate cancer cells. J Biol Chem.

[11] Chang AJ, Ortega FE, Riegler J, Madison DV, Krasnow MA (2015). Oxygen regulation of breathing through an olfactory receptor activated by lactate. Nature.

[12] Teixeira CF, Zamuner SR, Zuliani JP, Fernandes CM, Cruz-Hofling MA, Fernandes I (2003). Neutrophils do not contribute to local tissue damage, but play a key role in skeletal muscle regeneration, in mice injected with Bothrops asper snake venom. Muscle Nerve.

[13] Chazaud B, Sonnet C, Lafuste P, Bassez G, Rimaniol AC, Poron F (2003). Satellite cells attract monocytes and use macrophages as a support to escape apoptosis and enhance muscle growth. J Cell Biol.

[14] Murphy MM, Lawson JA, Mathew SJ, Hutcheson DA, Kardon G (2011). Satellite cells, connective tissue fibroblasts and their interactions are crucial for muscle regeneration. Development.

[15] Moss FP, Leblond CP (1971). Satellite cells as the source of nuclei in muscles of growing rats. Anat Rec.

[16] Crawford GE, Faulkner JA, Crosbie RH, Campbell KP, Froehner SC, Chamberlain JS (2000). Assembly of the dystrophin-associated protein complex does not require the dystrophin COOH-terminal domain. J Cell Biol.

[17] Katada S, Nakagawa T, Kataoka H, Touhara K (2003). Odorant response assays for a heterologously expressed olfactory receptor. Biochem Biophys Res Commun.

[18] Muscat GE, Kedes L (1987). Multiple 5′-flanking regions of the human alpha-skeletal actin gene synergistically modulate muscle-specific expression. Mol Cell Biol.

[19] Livak KJ, Schmittgen TD (2001). Analysis of relative gene expression data using real-time quantitative PCR and the 2(−delta delta C(T)) method. Methods.

[20] Pichavant C, Pavlath GK (2014). Incidence and severity of myofiber branching with regeneration and aging. Skelet Muscle.

[21] Chan S, Head SI (2011). The role of branched fibres in the pathogenesis of Duchenne muscular dystrophy. Exp Physiol.

[22] Schmalbruch H (1976). The morphology of regeneration of skeletal muscles in the rat. Tissue Cell.

[23] Sadeh M, Czyewski K, Stern LZ (1985). Chronic myopathy induced by repeated bupivacaine injections. J Neurol Sci.

[24] Head SI, Houweling PJ, Chan S, Chen G, Hardeman EC (2014). Properties of regenerated mouse extensor digitorum longus muscle following notexin injury. Exp Physiol.

[25] McGeachie JK, Grounds MD, Partridge TA, Morgan JE (1993). Age-related changes in replication of myogenic cells in mdx mice: quantitative autoradiographic studies. J Neurol Sci.

[26] Roig M, Roma J, Fargas A, Munell F (2004). Longitudinal pathologic study of the gastrocnemius muscle group in mdx mice. Acta Neuropathol.

[27] Chan S, Head SI, Morley JW (2007). Branched fibers in dystrophic mdx muscle are associated with a loss of force following lengthening contractions. Am J Physiol Cell Physiol.

[28] Hernandez-Ochoa EO, Pratt SJ, Garcia-Pelagio KP, Schneider MF, Lovering RM. Disruption of action potential and calcium signaling properties in malformed myofibers from dystrophin-deficient mice. Physiological reports. 2015;3(4). doi:10.14814/phy2.12366. 10.14814/phy2.12366 PMC4425971 25907787

[29] Fukuda N, Yomogida K, Okabe M, Touhara K (2004). Functional characterization of a mouse testicular olfactory receptor and its role in chemosensing and in regulation of sperm motility. J Cell Sci.

[30] Reynolds JG, McCalmon SA, Donaghey JA, Naya FJ (2008). Deregulated protein kinase A signaling and myospryn expression in muscular dystrophy. J Biol Chem.

[31] Jiang C, Wen Y, Kuroda K, Hannon K, Rudnicki MA, Kuang S (2014). Notch signaling deficiency underlies age-dependent depletion of satellite cells in muscular dystrophy. Dis Model Mech.

[32] Benedetti S, Cossu G, Tedesco FS, Templeton NS (2015). Gene and cell therapies for muscular dystrophies. Gene and cell therapy: therapeutic mechanisms and strategies.

[33] Guiraud S, Chen H, Burns DT, Davies KE. Advances in genetic therapeutic strategies for Duchenne muscular dystrophy. Experimental physiology. 2015. doi:10.1113/EP085308 10.1113/EP085308 PMC4973818 26140505

[34] Spehr M, Gisselmann G, Poplawski A, Riffell JA, Wetzel CH, Zimmer RK (2003). Identification of a testicular odorant receptor mediating human sperm chemotaxis. Science.

[35] Pluznick JL, Zou DJ, Zhang X, Yan Q, Rodriguez-Gil DJ, Eisner C (2009). Functional expression of the olfactory signaling system in the kidney. Proc Natl Acad Sci U S A.

[36] Serizawa S, Miyamichi K, Takeuchi H, Yamagishi Y, Suzuki M, Sakano H (2006). A neuronal identity code for the odorant receptor-specific and activity-dependent axon sorting. Cell.

[37] Imai T, Suzuki M, Sakano H (2006). Odorant receptor-derived cAMP signals direct axonal targeting. Science.

[38] Robertson TA, Papadimitriou JM, Grounds MD (1993). Fusion of myogenic cells to the newly sealed region of damaged myofibres in skeletal muscle regeneration. Neuropathol Appl Neurobiol.

