# Supplementary figures and images for: Decrease of myofiber branching via muscle-specific expression of the olfactory receptor mOR23 in dystrophic muscle leads to protection against mechanical stress
Source: Skelet Muscle. 2016 Jan 21;6:2. doi: 10.1186/s13395-016-0077-7 (PMC4721043; doi:10.1186/s13395-016-0077-7)

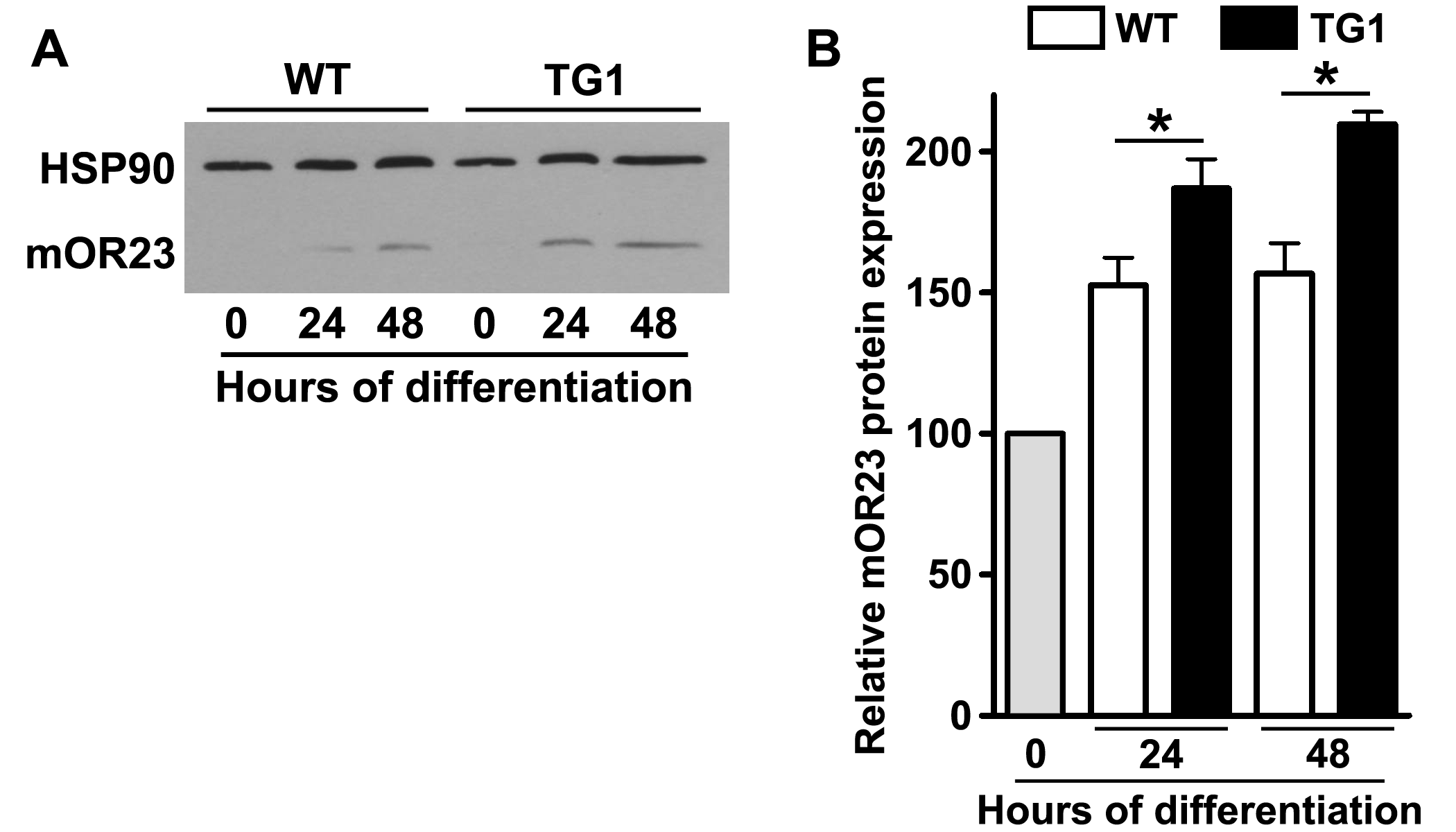

Supplement: Supplementary file 1 — mOR23 is over-expressed during myogenesis in TG1 myoblasts. (A) Immunoblot revealed that mOR23 levels increased after 48 h of myoblast differentiation in vitro but the levels of mOR23 were higher in TG1 muscle cells than in WT. (B) Quantification of mOR23 levels in differentiating muscle cells in vitro. Data are mean ± SEM; n = 3 with *p < 0.05. [file 13395_2016_77_MOESM1_ESM.tif]

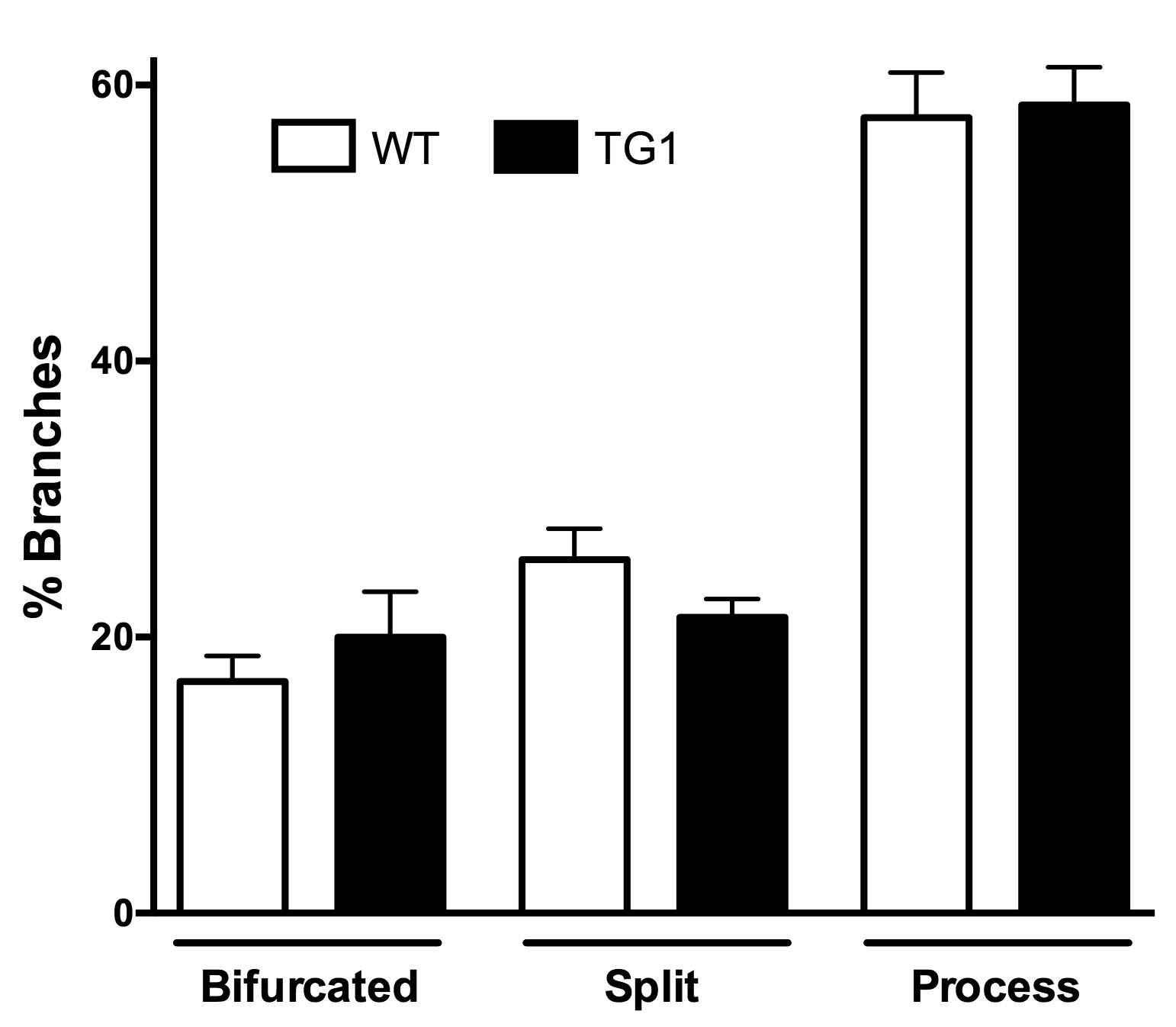

Supplement: Supplementary file 2 — Quantification of branch types in WT and TG1. No significant difference was observed between WT and TG1 myofibers for the distribution of the bifurcated, split, and process branch types 3 weeks after muscle injury. n = 27–88 branched myofibers isolated per mouse. Data are mean ± SEM and n = 4–6 mice for each genotype. [file 13395_2016_77_MOESM2_ESM.tiff]

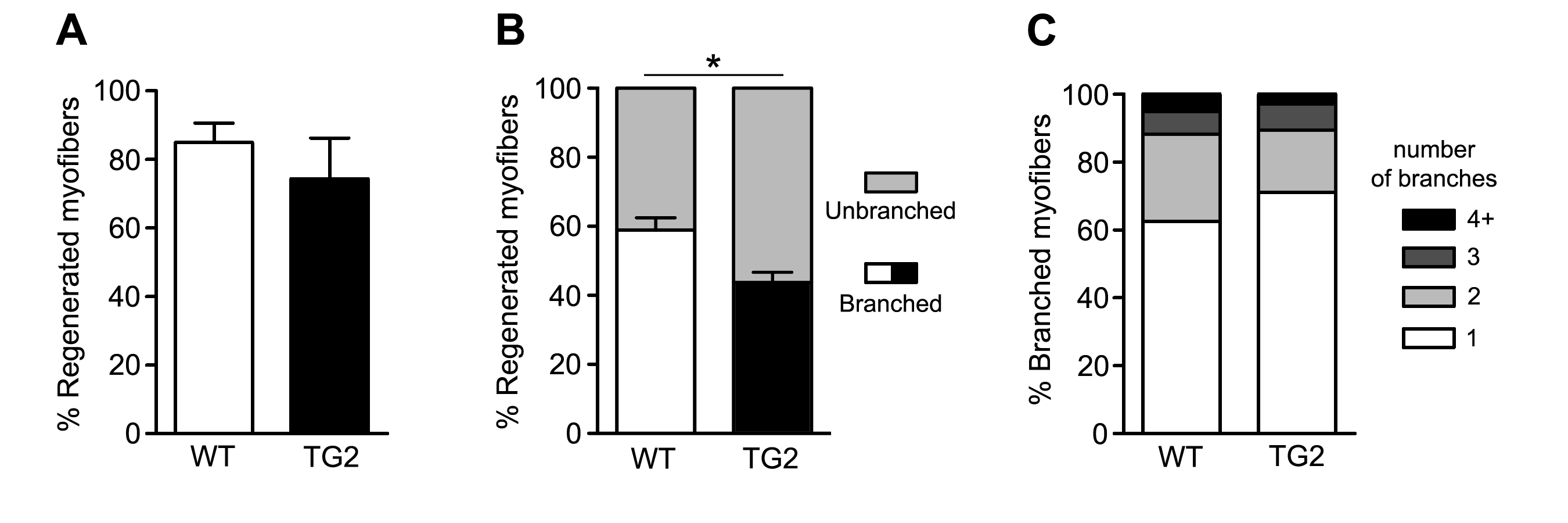

Supplement: Supplementary file 3 — Myofiber branching is also decreased in mOR23 transgenic mouse line 2 after muscle regeneration. (A) The percentage of regenerated myofibers in gastrocnemius muscles was not significantly different in WT and TG2 mice 3 weeks after muscle injury but TG2 muscles had significantly fewer branched regenerated myofibers than WT muscles (B). (C) The number of branches per branched myofiber was not significantly decreased in injured TG muscles compared to WT muscles (chi-square = 2.51, df = 3). n = 108–196 myofibers isolated per genotype and mouse. Data are mean ± SEM and n = 4 mice for each genotype with *p < 0.05. [file 13395_2016_77_MOESM3_ESM.tif]

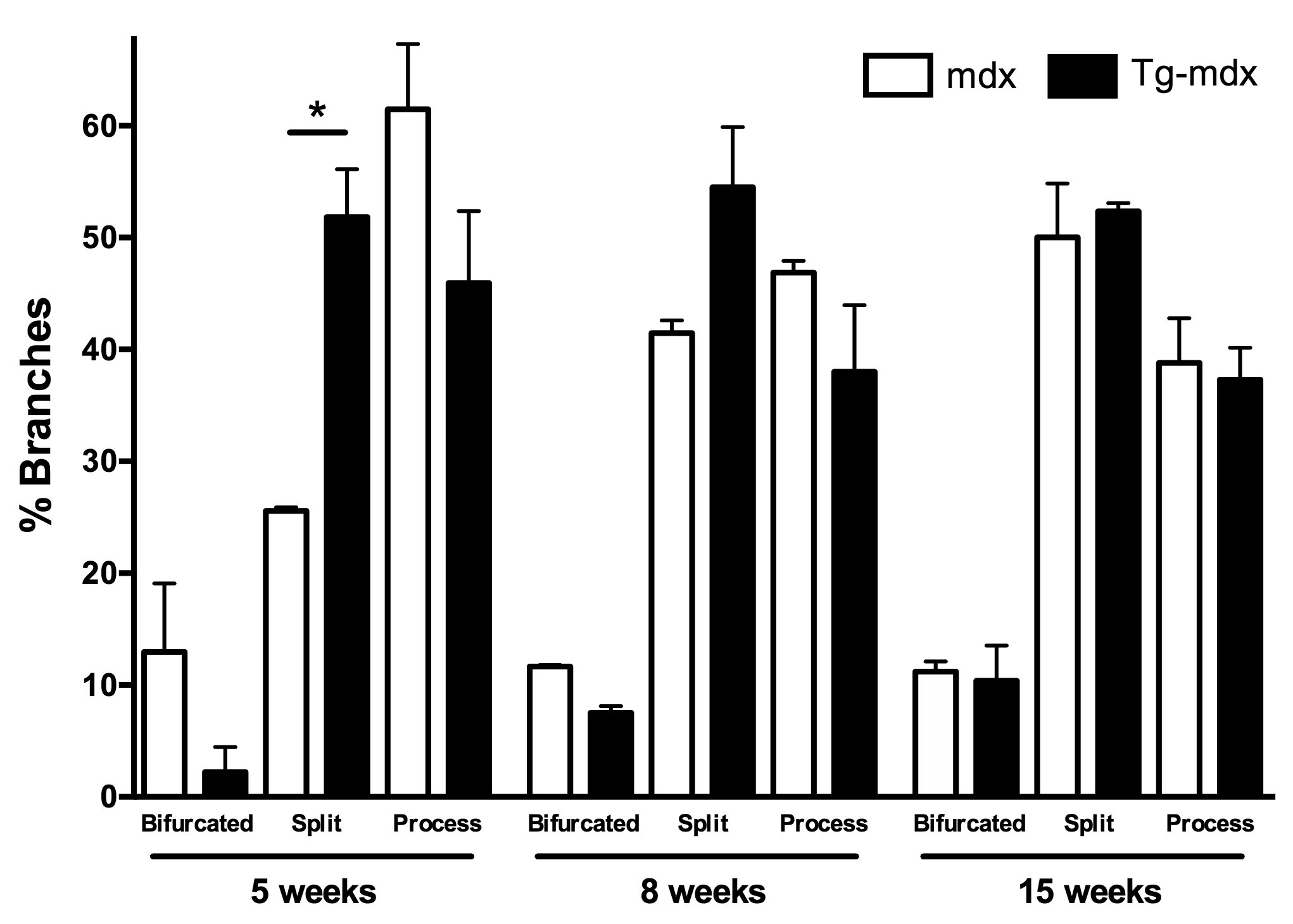

Supplement: Supplementary file 4 — Quantification of branch types in mdx and Tg-mdx. Except for a significant increase of the split branch type in mdx compared to Tg-mdx at 5 weeks of age, the types of branches did not differ between mdx and Tg-mdx. n = 7–64 branched myofibers isolated per age and mouse. Data are mean ± SEM and n = 3–4 for each genotype at each age with *p < 0.05. [file 13395_2016_77_MOESM4_ESM.tiff]
